# Supplementary material for: Viscoelasticity of Liposomal Dispersions
Source: Nanomaterials (Basel). 2023 Aug 15;13(16):2340. doi: 10.3390/nano13162340 (PMC10459094; doi:10.3390/nano13162340)
Supplement: Supplementary file 1 [file nanomaterials-13-02340-s001.zip › nanomaterials-2504719-supplementary.pdf]

Viscoelasticity of Liposomal Dispersions

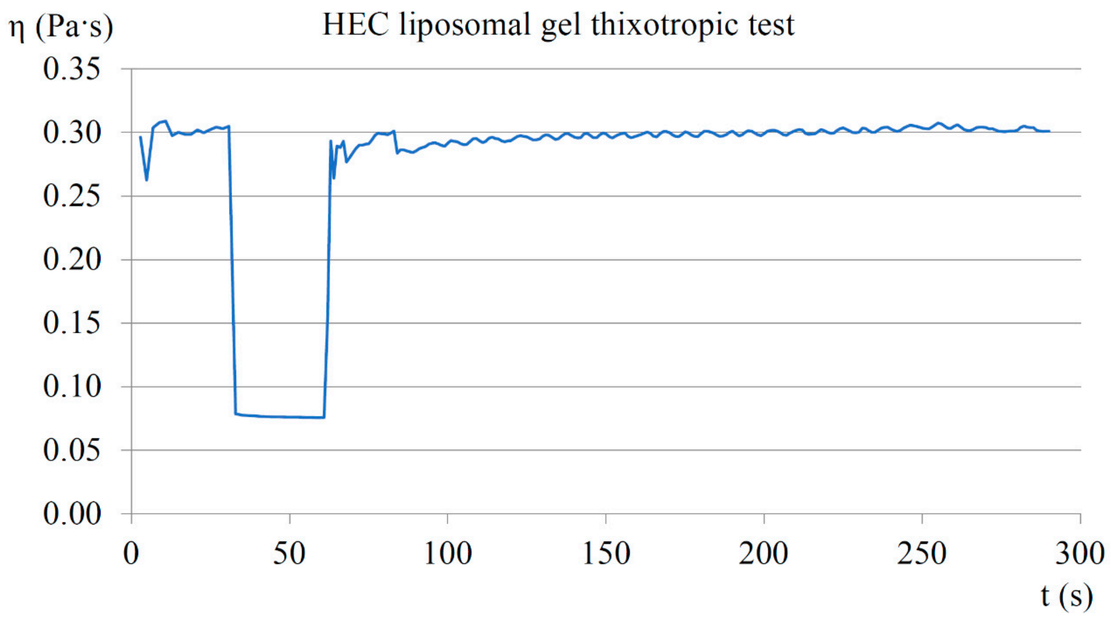

Figure S1. Thixotropic test of HEC liposomal gel

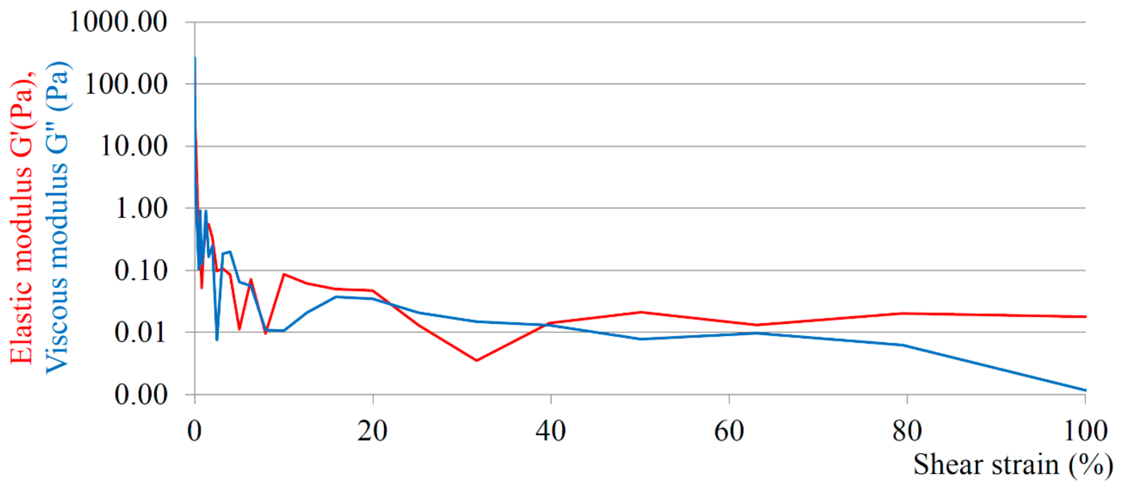

Figure S2. Amplitude sweep test of the sample LEC MLV (10.0 mg/ml)

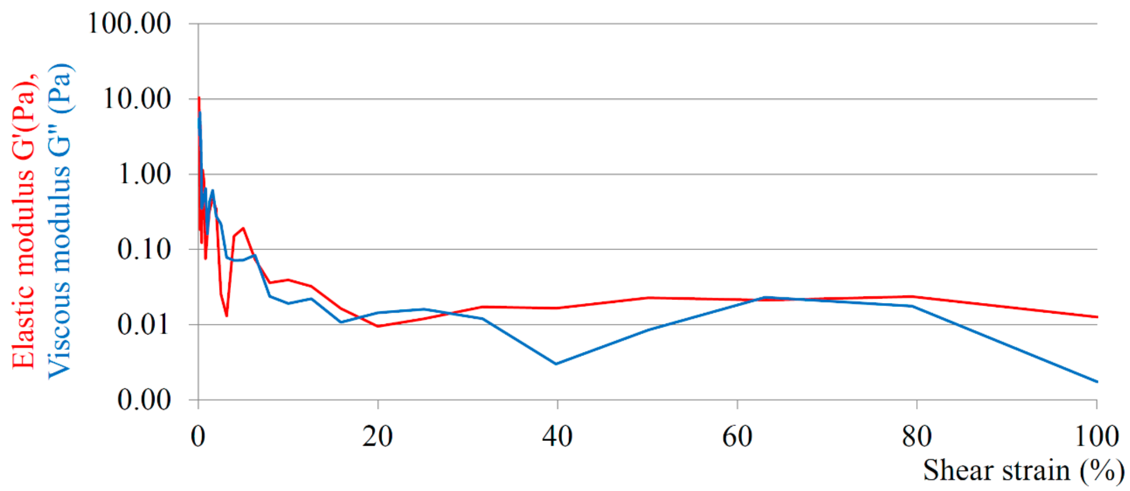

Figure S3. Amplitude sweep test of the sample LEC MLV (7.0 mg/ml)

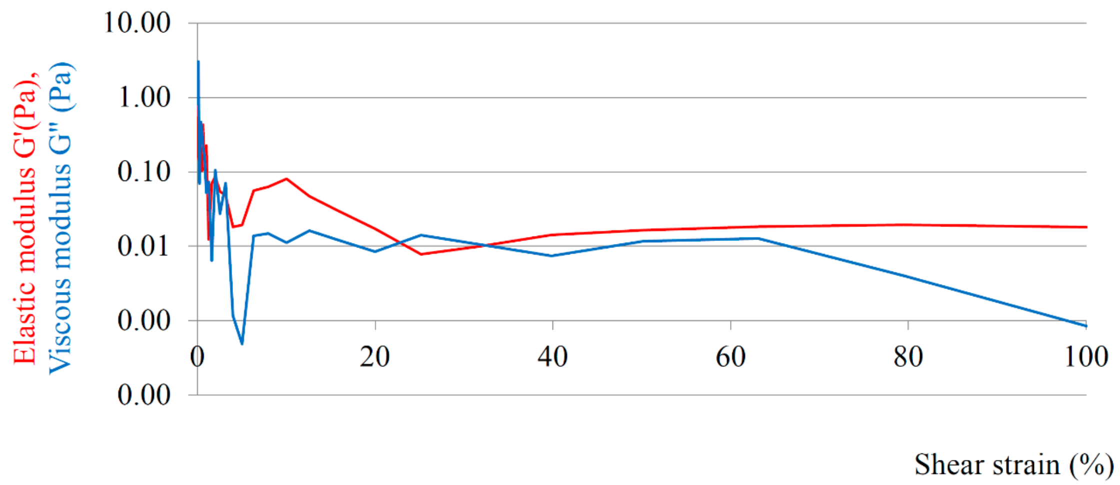

Figure S4. Amplitude sweep test of the sample LEC RSV (10.0 mg/ml)

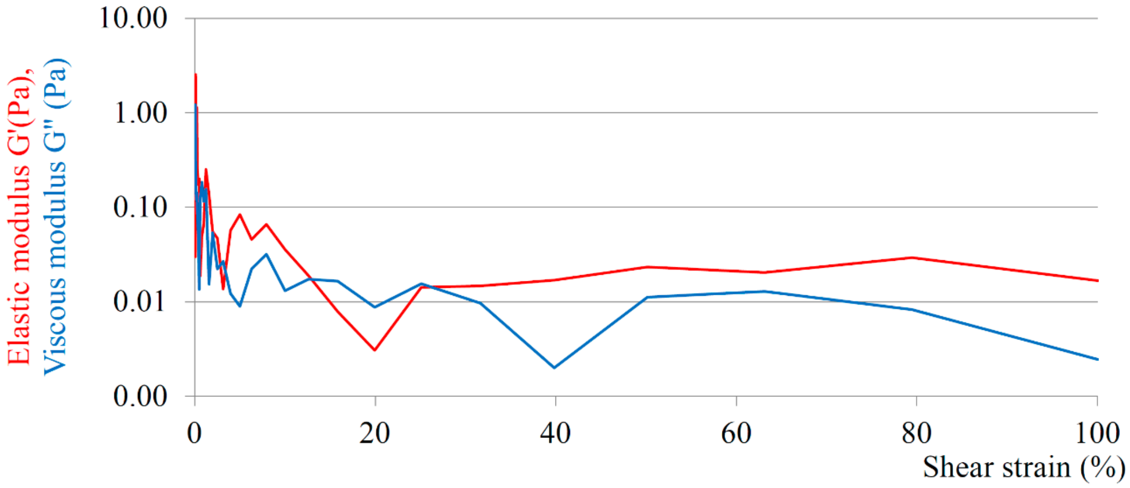

Figure S5. Amplitude sweep test of the sample LEC-CHOL (70/30) MLV (10.0 mg/ml)
